# Design and research of a passively mixed microfluidic chip for copper ion detection

**DOI:** 10.1371/journal.pone.0343203

**Published:** 2026-02-18

**Authors:** Yuxuan Geng, Longjiang Song, Junfei Wu, Wenjie Zhao, Ping Fu, Yanyong Liu, Luning Jia, Yalin Yuan

**Affiliations:** College of Mechanical and Electrical Engineering, Qingdao University of Science and Technology, Qingdao, Shandong, China; Children's Hospital of Orange County, UNITED STATES OF AMERICA

## Abstract

At the micro-scale channel dimensions and relatively low Reynolds numbers, fluids can only mix through diffusion in a laminar flow state. This dependence on molecular diffusion significantly hinders the mixing performance of microfluidic chips. To address this issue and promote the application of microfluidic technology in the detection of heavy metal ions, we propose a high-performance microfluidic chip with variable cross-sectional channels based on passive mixing. By setting bias centrifugal bends and linear flow channels with periodic diameter changes, the mixing efficiency of the microfluidic chip has been significantly improved. To verify the theoretical mixing effect, we set up eight groups of different Reynolds number conditions for the microfluidic chip and simulated the fluid flow in laminar state. Through analyzing the simulation cloud diagrams and the mass fraction mixing index, it was found that when the Reynolds number was 0.5, the mixing efficiency of the microfluidic chip reached the optimal state, with a mass fraction mixing index of 0.9998, and the pressure drop was only 0.1502 Pa, which was higher than the mixing efficiency of similar chips under the same conditions. Using 3D printing technology to fabricate the microfluidic chip and conducting characterization analysis. To verify the actual mixing effect, a colorimetric mixing experiment was set up, and a visual mixing effect analysis of the chip was conducted. Through ICP-MS for copper ion detection experiments, three control experiments were set up to conduct a data-driven mixed effect comparison analysis of the chip. After verification, the overall and local mixing effects of the microfluidic chip were highly consistent with the simulation results under the same conditions, and the detection value of the mixed solution was 101.99% of the completely mixed solution, showing good consistency. Therefore, this chip has excellent mixing performance and is conducive to promoting the application of passive microfluidic chips in fields such as heavy metal detection.

## Introduction

With the rapid development of modern industry, the phenomenon of discharging heavy metal wastewater into the sea has become increasingly common [1]. Traditional detection methods usually require on-site sampling and then sending the samples back to the laboratory for analysis, such as inductively coupled plasma atomic emission spectroscopy [2–4], atomic absorption spectrophotometry [5–7], electrochemical methods [8–10], cold vapor atomic fluorescence spectroscopy (CV-AFS) [11], surface-enhanced Raman scattering spectroscopy (SERS) [12], and aptamer sensor technology [13,14]. These methods all require testing in the laboratory, resulting in relatively low accuracy of the detection data. To achieve precise detection of heavy metals and real-time monitoring of the marine environment, the importance of portable on-site analysis technologies is increasingly prominent. Seawater heavy metal detection equipment based on microfluidic technology can effectively improve detection efficiency, enhance detection accuracy, simplify the detection process, reduce detection costs, and support online monitoring.

At present, analytical and monitoring instruments based on microfluidic technology have achieved portability, miniaturization, low energy consumption and integrated automation, meeting the requirements for heavy metal detection and monitoring. Experts and scholars at home and abroad have widely applied microfluidic technology to detect and analyze heavy metals and other substances in different water bodies, achieving fruitful results. Shariaat et al. [15] developed a visible and distinguishable microfluidic chip. When samples containing Hg2 + come into contact with the color-developing reagent, the color will immediately change. The Tabani team [16] developed a method combining double-gel electrode extraction and microfluidic analysis equipment, which can simultaneously detect Cr4+ and Cr3+ in water. Zhang et al. [17] developed an infinite-pattern microfluidic sensor, which verified its accuracy in the detection of Fe2 + , Ni2 + , and Cu2 + . Some scholars also regulate the flow channel structure of microfluidic chips through methods such as diversion, distortion, or the installation of guide columns, in order to enhance the mixing efficiency in laminar flow conditions. Ma et al. [18] designed a 3D-printed microfluidic device combined with screen-printed electrodes based on the thermal capillary convection mechanism. The advantage of this design lies in its high sensitivity, rapidity, portability, and the absence of the need for separate pre-enrichment. It can efficiently achieve on-site detection of Pb2 + , and the test results are highly consistent with ICP-MS. Wisarut et al. [19] designed a microfluidic chip using variable cross-sectional channels and passive mixing geometric units. The advantage of this design is its simple structure, no energy consumption, high mixing efficiency, and stability, which can efficiently promote copper ion detection. This fully demonstrates the broad prospects of passive mixing in the field of heavy metal detection. Although researchers have applied microfluidic technology to heavy metal detection, some scholars have also conducted research on the structure of the microfluidic chip itself, but no effective integration of the two has been achieved. Therefore, there are still three main problems in the application of microfluidic technology in heavy metal detection at present: neglecting the mixing performance of the chip, the need for improvement in the mixing performance, and the lack of integration of analysis and verification.

Based on this, this research aims to address the key challenge of poor mixing efficiency of the microfluidic platform under low Reynolds numbers (Re < 1).To solve this problem, we designed a high-performance microfluidic chip with a variable cross-sectional flow channel based on the principle of passive mixing. Compared with active mixing, passive mixing does not require external driving components. It has a compact structure, is easy to operate, can effectively reduce the manufacturing cost and system complexity of the chip, and avoids possible interference or damage to the detection sample caused by external fields. Thus, while ensuring the mixing performance, it improves the reliability and integration of the entire detection system.To overcome this challenge, through simulation experiments, we conducted simulations of the mixing effect of microfluidic chips under different Reynolds numbers, and deeply explored the mixing characteristics of the mixing diagrams, streamline diagrams, and local cloud diagrams. Color comparison mixing experiments were carried out for visual verification, and the actual mixing performance of the microfluidic chips under different conditions was studied. After considering various factors comprehensively, the ICP-MS method was ultimately selected to detect copper ions in the solution, achieving data-based verification of the mixing performance of the microfluidic chips. Through simulation analysis, color mixture experiments and copper ion detection experiments, the excellent mixing performance of this microfluidic chip was verified, providing theoretical basis and practical support for the application of microfluidic technology in the field of heavy metal detection.

## Microfluidic chip structure design and simulation

### Microfluidic chip structural design

The continuous “S” type microfluidic chip is optimized based on the “S” type microfluidic chip structure. The specific dimensions are shown in Table 1. The flow channels consist of 6 sets of eccentric centrifugal bends and 5 straight channels with diameters that change periodically. The inlet channel adopts a symmetrical distribution design to ensure the stability of the initial liquid flow state. The mixed liquid is discharged from a single outlet in the rear flow field area of the chip. The outlet channel adopts a gradually expanding structure design, which can balance the flow velocity and reduce pressure loss.

**Table 1:** Parameters of Continuous “S” Shape Microfluidic Chip.

Structure	Dimension parameter
Overall Size	40mm*20 mm*2 mm
Centrifugal bend radius (large)	2.4mm
Centrifugal bend radius (small)	1.2mm
Maximum diameter of the linear flow channel	1.0mm
Minimum diameter of the linear flow channel	0.4mm
Diameter of the diversion column	0.36mm

**Fig 1:**
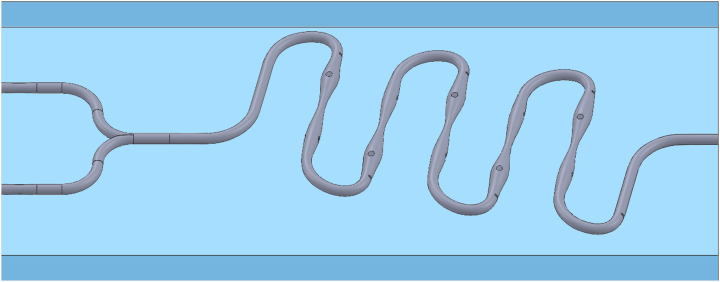
Continuous “S” shaped microfluidic chip.

The overall structure of the microfluidic chip is shown in Fig 1. The radius of the centrifugal bend in the front half is 2.4 millimeters, which is twice that of the rear half. This asymmetric centrifugal bend with a bias enables the fluid to flow laterally and generate secondary flow within the channel [20,21]. At the same time, in the micro-mixing process, this design can minimize the impact of excessively high pressure drop on the fluid velocity to the greatest extent [22], and reduce the overall size of the microfluidic chip. At the centrifugal bend, due to the effect of inertial centrifugal force, the denser solid particles will migrate to the outer side of the channel. Therefore, a baffle is embedded at the outer end of the centrifugal bend to filter the solid particles in the seawater using the inertial focusing effect [23].

Among the five linear flow channels of the microfluidic chip, the cross-sectional dimensions of the channels exhibit periodic changes. The maximum diameter is 1.0 millimeter, and the minimum diameter is 0.4 millimeter. The cross-section of each flow channel stage undergoes two gradual changes from coarse to fine. According to Poiseuille’s law [24], this periodic change in cross-section can trigger fluid vortices and secondary flows [25]. The transverse and longitudinal elliptical guide columns set at the widest part, combined with the periodic changes of the flow channels, significantly enhance the convective mixing effect of the fluid inside the microfluidic chip [26].

### Basic control equations for microfluidic chip mixing

Due to the small scale and low velocity of the fluid in the microfluidic chip, it is in a laminar flow state. Generally, the fluid is considered to be an incompressible Newtonian fluid and the energy transfer during the mixing process is not taken into account. In this study, eight sets of simulation Reynolds numbers Re were selected, namely 0.5, 1, 5, 10, 20, 50, 100, and 200, which are far below the upper critical value of the laminar flow state [27] (it is generally believed that when Re < 2300, the fluid is in a laminar flow state). Therefore, all the simulations in this study are in a laminar flow state. For the fluid flow in this state, the flow field can be solved only by using the mass conservation equation and the motion equation.

The fluid flow within the microfluidic chip complies with the principle of mass conservation. The fluid characteristics within the chip can be inferred using the calculation formula of the Reynolds number [28].


$$Re=\frac{\rho vd}{\mu }$$ (1)

In the formula: *ρ* represents the density of the fluid, *v* represents the velocity of the fluid, *μ* represents the viscosity coefficient, *d* represents the characteristic length of the pipeline.

Based on the incompressibility of the fluid, we obtain ∂/∂t = 0. When ignoring the friction force f, in order to solve the fluid motion problem in the microfluidic chip, the following two equations need to be applied: the fluid continuity equation and the three-dimensional Navier-Stokes equation [29].


$$\begin{array}{c} \nabla u=\text{0} \end{array}$$ (2)

$$\begin{array}{c} \rho \frac{\partial u}{\partial t}+\rho (u\cdot \nabla u)=\nabla \cdot \left[-pI+\mu \left(\nabla u+\nabla u^{T}\right)\right]+F_{b} \end{array}$$ (3)

Fick’s law pertains to mass transfer phenomena that do not occur through fluid convection. It is used to describe the relationship between the mass transfer flux and the concentration gradient during molecular diffusion, including Fick’s First Law and Fick’s Second Law. The first law is applicable to the diffusion of substances under stable conditions (∂c/∂t = 0, the substance concentration and concentration gradient only depend on position). Fick’s First Law is expressed in three-dimensional scale as Equation (4). Here, J represents the diffusion flux of substances through a unit cross-section perpendicular to the diffusion direction (referred to as diffusion flux), which is proportional to the concentration gradient at that cross-section. The greater the concentration gradient, the greater the diffusion flux. The negative sign indicates that the direction of substance diffusion is opposite to the direction of the concentration gradient; *c* (mol/m^3^) is the concentration of the solute in the solution; *D* (m^2^/s) represents the diffusion coefficient of the substance, which describes the speed of substance diffusion. Its magnitude is not only related to the type of solute but also to the solvent and temperature and other conditions [30].


$$\vec{J}=\vec{ı}J_{x}+\vec{J}J_{y}+\vec{w}J_{z}=-D\left(\frac{\partial c}{\partial x}\vec{ı}+\frac{\partial c}{\partial x}\vec{J}+\frac{\partial c}{\partial x}\vec{w}\right)$$ (4)

In the research on fluid mixing in microfluidic chips, the mass fraction mixing index (M) is an indicator used to quantify the mixing effect. Its core formula is usually based on the uniformity of the concentration distribution of the target components (such as tracers or solutes) in the fluid.


$$M=\text{1}-\frac{\sqrt{\frac{\text{1}}{N}\sum_{i=\text{1}}^{N}{\left(Y_{i}-\bar{Y}\right)}^{\text{2}}}}{\bar{Y}}$$ (5)

*Y*_*i*_ represents the mass fraction of a certain component in the i-th grid cell. $\bar{Y\;}$ is the average mass fraction of this component at the outlet. *N* is the total number of grid cells at the outlet. The closer *M* is to 1, the more uniform the mixture is; the closer it is to 0, the less uniform the mixture is.

Generally speaking, in micro-scale systems such as microfluidic chips, the fluid is in a low Re (laminar flow) state, and special flow channel designs (such as serpentine, contraction-expansion structures) need to be combined with fluid mechanics characteristics to enhance the convective mixing of the fluid, in order to supplement the final mixing efficiency of diffusion mixing and achieve an efficient and controllable micro-mixing process of the fluid. In this study, the mass fraction mixing index (M) was selected as the parameter index for analyzing the mixing effect of microfluidic chips, and together with the simulation simulation cloud diagram, they were used as the basis for testing the mixing effect.

### Meshing and grid independence verification

The continuous “S”-shaped microfluidic chip model was imported into ANSYS® Fluent 2021 R2 software, and the fluid domain was extracted. The inlet, outlet, and wall surfaces were defined. Since the aim was to analyze the influence of structures such as the “S”-shaped channel and the flow guide column on the mixing performance, to avoid defects in element skewness, hexahedral grids were selected for meshing the continuous “S”-shaped microfluidic chip model. To eliminate the potential impact of the number of grids on the accuracy of the numerical simulation results, grid independence verification was conducted. In this study, by systematically changing the global grid size, three sets of grids with different densities from coarse to fine were generated, and the differences in the mixing efficiency at key monitoring positions (such as the outlet cross-section) or specific flow field parameters (such as pressure drop and velocity distribution) were compared. The results indicated that when the number of grids reached a certain level, further grid refinement had an impact on the above key results less than the preset threshold (2%), and it could be considered that the calculation results were basically not affected by the number of grids. Finally, a grid scheme that balanced calculation accuracy and efficiency was selected for subsequent analysis.

**Fig 2:**
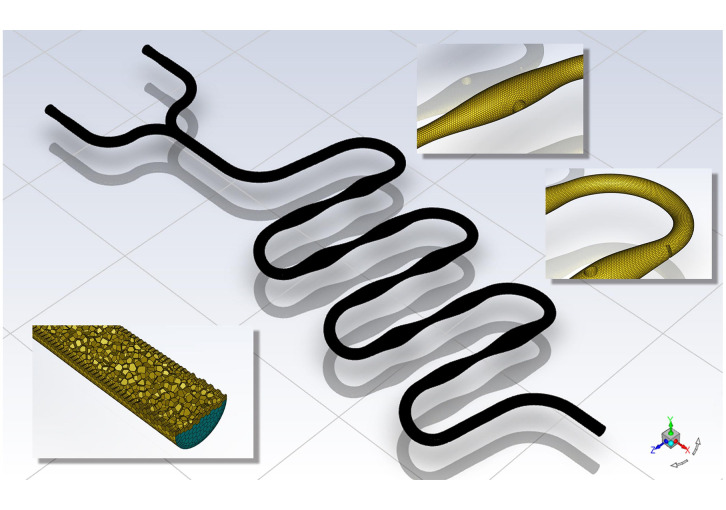
Grid formation of continuous “S” shape microfluidic chip.

The surface grid number of the continuous “S”-shaped microfluidic chip was 3,086,961, and the volume grid number was 605,247, as shown in Fig 2. The overall grid division was fine and compact. The lower left is the cross-sectional view of the volume grid, and the upper right is the local view of the surface grid.

### Analysis of simulation results

#### Overall analysis of simulation modeling.

To study the mixing performance of a high-performance variable-section flow channel microfluidic chip based on passive mixing, we used the component transport model in ANSYS®Fluent 2021 R2 software to simulate the flow velocity field of the chip. The fluid mass diffusion rate is 1e-10 m²/s, the density is set at 1000 kg/m³, the viscosity is 0.001003 kg/m*s, and the molecular weight is 18.0152 kg/mol. By setting the Reynolds number Re to 0.5, 1, 5, 10, 20, 50, 100 and 200, a total of 8 simulation experiments were conducted to verify the mixing effect of the chip under different working conditions. The parameters used in the experiment were the general mass fraction mixing index M and pressure drop ΔP.

**Fig 3:**
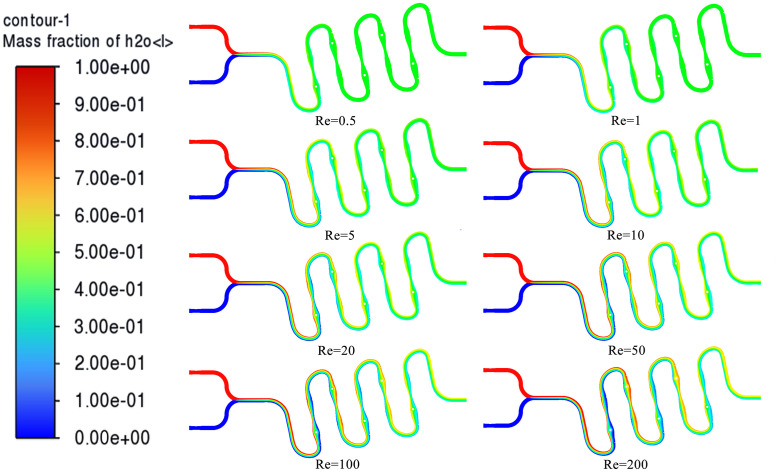
Simulation of continuous “S” shape microfluidic chip for mixed cloud visualization.

As shown in Fig 3, in the 8 simulation experiments, the color difference of the continuous “S” type microfluidic chip mixing cloud graph increased with the increase of Re. This indicates that the overall mixing effect of the chip gradually weakened as Re increased. When Re = 0.5 and Re = 1, the two fluid intersection stages of the continuous “S” type microfluidic chip showed obvious color differences, while the main part of the continuous “S” type flow channel presented uniform green color, indicating that when Re = 0.5 and Re = 1, the fluids achieved sufficient mixing inside the chip. When 5 < Re < 100, the areas with obvious color differences in the continuous “S” type flow channel increased with the increase of Re, and the laminar effect was clearly visible, indicating that as Re increased, the mixing time of the fluid inside the chip gradually shortened, and thus the distance required to achieve sufficient mixing increased accordingly [31,32]. When Re = 5 and Re = 10, the cloud graph at the outlet stage of the continuous “S” type microfluidic chip had no obvious color difference at this time, and the fluid still achieved basic mixing inside the chip; while when 20 < Re < 100, the proportion of yellow in the upper cloud graph at the outlet stage of the continuous “S” type microfluidic chip continuously increased, indicating that in these 3 simulation experiments, the mixing effect of the fluid inside the chip was not sufficient and gradually weakened. When Re = 200, the simulation cloud graph in the “entry intersection-continuous “S” type flow channel-outlet stage” all showed obvious color differences and the degree of color difference gradually weakened, indicating that although the fluid was mixed inside the chip through the action of the flow channel structure, it did not achieve sufficient mixing.

**Fig 4:**
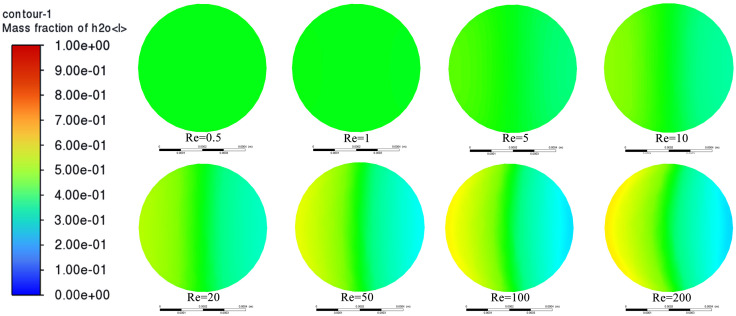
Mixing cloud diagrams at the chip outlet under different Reynolds numbers.

To further study the overall mixing performance of the continuous “S” type microfluidic chip, the mixed cloud diagrams at the simulation outlet of 8 groups were analyzed. As shown in Fig 4, when Re = 0.5 and Re = 1, the mixed cloud diagram at the outlet shows a uniform green color, indicating that the two fluids have reached a fully mixed state after flowing through the continuous “S” type microfluidic chip. When Re = 5, slight color differences begin to appear on both sides of the mixed cloud diagram, indicating that the two fluids have basically achieved a fully mixed state under this condition. When Re = 10, the color difference of the mixed cloud diagram is higher than that at Re = 5, but the color difference is still at a relatively low level. At this time, the fluid still has a relatively ideal mixing effect. As Re continues to increase (10 < Re < 200), the color differences on both sides of the mixed cloud diagram gradually increase, and the uniform green area is gradually compressed, indicating that the mixing effect of the two fluids is not ideal and is showing a downward trend. In conclusion, when Re is relatively low (0.5 < Re < 10), the continuous “S” type flow channel structure can better achieve the mixing of the two fluids. When Re is relatively high (Re > 10), the induced effect of the continuous “S” type flow channel structure on the fluid cannot compensate for the mixing defect caused by high flow rate and short time, so it fails to present a good mixing effect [33].

**Fig 5:**
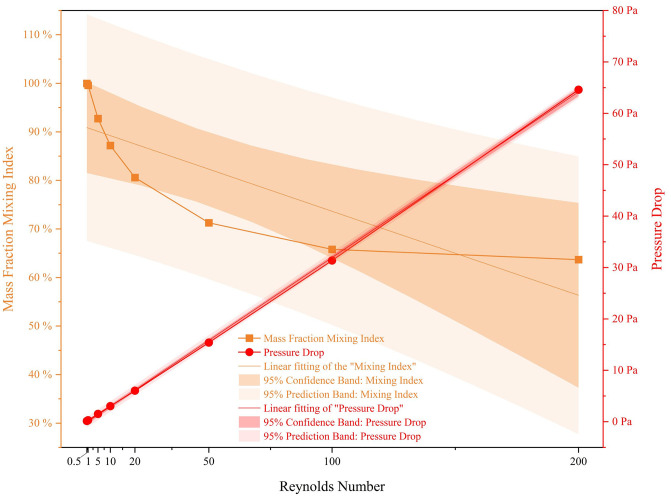
Curve of mixing index and pressure drop for the chip under different Reynolds numbers.

As shown in Table 2 and Fig 5, as the Re value keeps increasing, the overall quality fraction mixing index shows a downward trend and stabilizes after Re > 50, with a standard deviation of 0.1363. The highest quality fraction mixing index occurs in the group with Re = 0.5, with a value of 0.9998, and the corresponding pressure drop is 0.1502 Pa; the lowest quality fraction mixing index occurs in the group with Re = 200, with a value of 0.6369, and the corresponding pressure drop is 64.5804 Pa. The variation of pressure drop values is inversely proportional to Re, and it follows a clear linear pattern. This phenomenon occurs mainly because in micro-scale flow and under low Reynolds numbers (0.5 < Re < 200), the fluid is mainly in a laminar state and almost no turbulence is generated, avoiding the nonlinear change of the friction factor f under high Re conditions. The generation of pressure drop is due to the fact that in microfluidic chips, the fluid flows forward under the constraint of the channel, experiencing kinetic energy loss caused by the offset centrifugal bend and the linear flow channels with periodic diameter changes, as well as greater energy loss during convection.

**Table 2:** Parameters for the mixing effect of fluids in different states.

Group	Re	Initial injection flow rate (mm/s)	M	Standard Deviation(σ)	ΔP(Pa)
1	0.5	0.4179	0.9998	0.1363	0.1502
2	1	0.8358	0.9958		0.3018
3	5	4.1792	0.9274		1.5117
4	10	8.3583	0.8719		3.0294
5	20	16.7166	0.8059		6.0471
6	50	41.7917	0.7128		15.3929
7	100	83.5833	0.6579		31.3785
8	200	167.1666	0.6369		64.5804

#### Analysis of simulation flowcharts and local diagrams.

**Fig 6:**
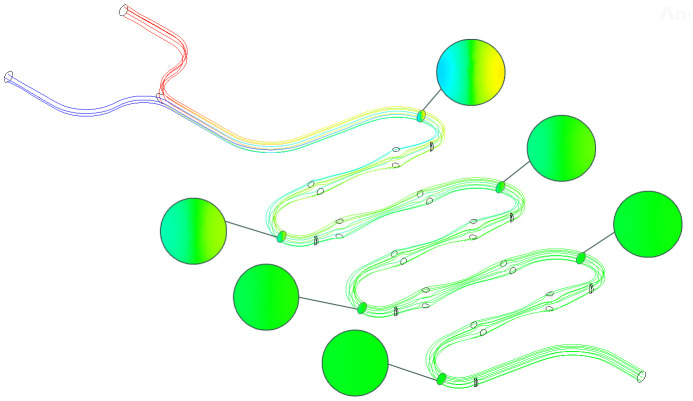
S-shaped continuous microfluidic chip flowline diagram and cross-sectional cloud diagram (Re = 1).

Due to the difficulty in achieving the experimental condition of Re = 0.5 in the actual environment, compared to this, when Re = 1, the fluid mixing effect is also quite excellent, and the influence of the chip structure on fluid mixing is more obvious. Therefore, this group was selected for analysis. As shown in Fig 6, six offset centrifugal bends in the middle section were extracted to obtain cross-sectional contour diagrams from distances of 16.7 mm, 19.1 mm, 23.7 mm, 26.2 mm, 30.8 mm, and 33.2 mm from the inlet. When the fluid was in the first cross-section of the flow path, the two fluids presented a clear laminar flow state. At the second cross-section, the fluid mixing effect was significantly improved, but there was still a small amount of fluid that failed to achieve full mixing on both sides. At the third cross-section, the overall contour diagram showed a good mixing effect, indicating that the fluid achieved basic mixing at this point. The mixing contour diagrams of the 4th, 5th, and 6th cross-sections all showed uniform green color, with no obvious color difference and could not be distinguished by the naked eye. Therefore, a data analysis based on the quality fraction mixing index was required.

As the fluid flowed through different groups of complex cross-sectional flow paths within the microfluidic chip, the quality fraction mixing index continuously increased. The quality fraction mixing indices of the six offset centrifugal bends in the middle section of the fluid were 0.6122, 0.8297, 0.9137, 0.9625, 0.9811, and 0.9918, and the quality fraction mixing index at the end of the chip was 0.9958. Compared to the quality fraction mixing index of the VSE-type microfluidic chip in the same conditions in the literature [34], it was 16.25% higher. Among them, the growth value of the quality fraction mixing index of the first offset centrifugal bend section was the highest, at 0.6122. This is because at the front end of the microfluidic chip, there is a longer smooth flow path, and the fluid undergoes diffusion mixing without turbulent disturbance in laminar flow state. The growth amplitudes of the quality fraction mixing indices of the latter 4 offset centrifugal bend sections compared to the first offset centrifugal bend section were 10.1241%, 5.3409%, 1.9325%, and 1.0906%, respectively. Among these, the growth amplitudes of the quality fraction mixing indices of the latter 3 offset centrifugal bend sections were all below 10%, which is due to the continuous advancement of the mixing process, the increase in fluid mixing degree, the decrease in concentration gradient, and the weakening of the diffusion driving force in the subsequent groups of the chip, resulting in a slower growth rate of the quality fraction mixing index [35].

Under the conditions of Re = 0.5 and Re = 1, the final quality fraction mixing indices of the continuous “S” type microfluidic chip were 0.9998 and 0.9958, respectively. Therefore, the mixing performance of the continuous “S” type microfluidic chip can meet the experimental requirements for the detection of heavy metals in seawater.

## 3D printing microfluidic chips and performance characterization verification

The precise fabrication of microfluidic chips is the key to the success of the experiment. Designing and processing microfluidic chips with specific flow channel structures is the core method for achieving efficient fluid mixing and precise control, and it is also a crucial step to ensure the accuracy and reliability of subsequent observation and measurement data. Based on the analysis of simulation models, 3D printing technology-DLP is used to fabricate microfluidic chips, and high-definition electron microscopes are employed to conduct performance characterization analysis and verification of the chips.

### 3D printing equipment and materials

**Fig 7:**
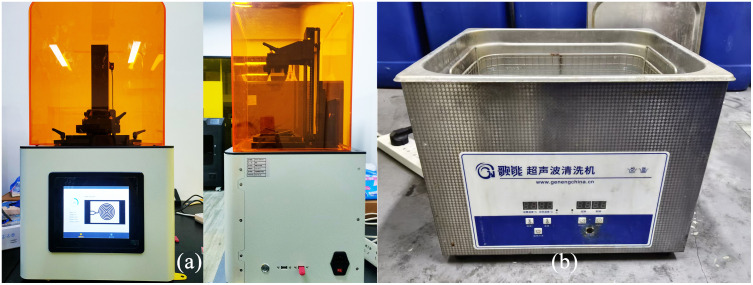
Microfluidic Chip Preparation Equipment.

The 3D printing equipment used is a resin 3D printer purchased from Borico Company, as shown in Fig 7(a). The printing material selected is high-precision resin. The specific parameters of the printing equipment and materials are presented in Table 3.

**Table 3:** Parameters of 3D Printing Equipment and Materials.

Equipment/ Materials	Name	Specific Parameters
Resin 3D Printer	Model	BL3D-50-P2
	Resolution	1920*1080
	Horizontal Resolution	25µm
	Layer Thickness	10-100µm
	Printing Accuracy	0.02mm
	Construction Speed	10-30 mm/h
	Layer Curing Time	3s
High-Precision Resin	Model	BPHP-01
	Hardness	86-88HB
	Bending Strength	95-100MPa
	Tensile Strength	55-65MPa
	Breakage Elongation Rate	5-8%
	Density	1.05-1.15g/cm^3^@ 25°C
	Viscosity	250-300 cps @ 25°C
	Heat Shrinkage Temperature	100-110°C

### Preparation steps of microfluidic chips

The microfluidic chip is fabricated using 3D printing technology based on Digital Light Processing (DLP). This process mainly consists of five steps: importing and preprocessing the digital model, slicing the 3D model and planning the printing path, DLP photopolymerization printing, cleaning after printing to remove un-solidified resin, and purifying the internal channels of the device with high-pressure gas. The specific operations and principles of each step are as follows:

#### Importing the digital model and pre-processing.

Firstly, the three-dimensional model of the microfluidic chip constructed in SolidWorks software is exported as a “.STL” format file and imported into the dedicated slicing software for the DLP printer. At this stage, pre-processing operations such as optimizing the printing orientation and automatically/manually adding support structures need to be performed. Adjust the layout of the chip’s printing orientation to reduce the step effect during the printing process and ensure the structural integrity and surface quality of the device’s functional plane.

#### Slicing of the three-dimensional model and setting of printing parameters.

The slicing software discretizes the imported three-dimensional digital model along the Z-axis (construction direction) layer by layer to generate a series of two-dimensional digital slice files representing the cross-sectional contours of each layer. At the same time, based on the printing performance of the BL3D-50-P2 device and the characteristics of the BPHP-01 resin, the core process parameters that affect the printing quality are set. The parameter settings determine the curing thickness of each layer of resin, the shape fidelity, and the bonding strength between layers, which are the key to achieving high-precision molding of the microstructure.

#### DLP photopolymerization printing and forming.

During the printing stage, the build platform first descends to the bottom of the material tray, creating a thin layer of liquid photoinitiating resin between the platform and the optical transparent film at the bottom of the tray. Then, the DLP projector precisely projects the specific wavelength ultraviolet light pattern onto the resin layer. The resin in the irradiated area undergoes a selective photopolymerization reaction, transforming from liquid to solid and firmly bonding with the build platform or the previously cured part of the previous layer. After the layer is cured, the build platform is raised, the liquid resin is refreshed, and it descends again, repeating this process until the entire chip entity is accumulated layer by layer.

#### Cleaning after printing to remove un-cured resin.

At the beginning of the printing process, the surface of the chip entity and the microchannels inside it will be covered with a large amount of un-participating liquid resin. The device should be immediately immersed in high-purity alcohol (95% ethanol) and washed with 4 minutes of ultrasonic cleaning, as shown in Fig 7(b), using a Songneng brand ultrasonic cleaning machine. The cavitation effect produced by the ultrasonic wave in the liquid can effectively penetrate into the micro-micron channels and violently impact and disintegrate the residual resin, ensuring the initial cleaning of the complex flow channel network.

#### High-pressure gas-driven purification of the internal channels of the device.

After the liquid cleaning, there may still be a small amount of cleaning liquid and resin mixture remaining inside the chip. Therefore, a filtered, dried, and pressure-controlled gas source is used to apply a 4-minute high-pressure gas flow to the fluid inlet and outlet of the chip, thoroughly blowing out the liquid residues hidden in the complex structure of the chip channels, avoiding the blockage of the flow channels or affecting the optical transparency and biocompatibility of the chip.

### Preliminary observation of microfluidic chip products

**Fig 8:**
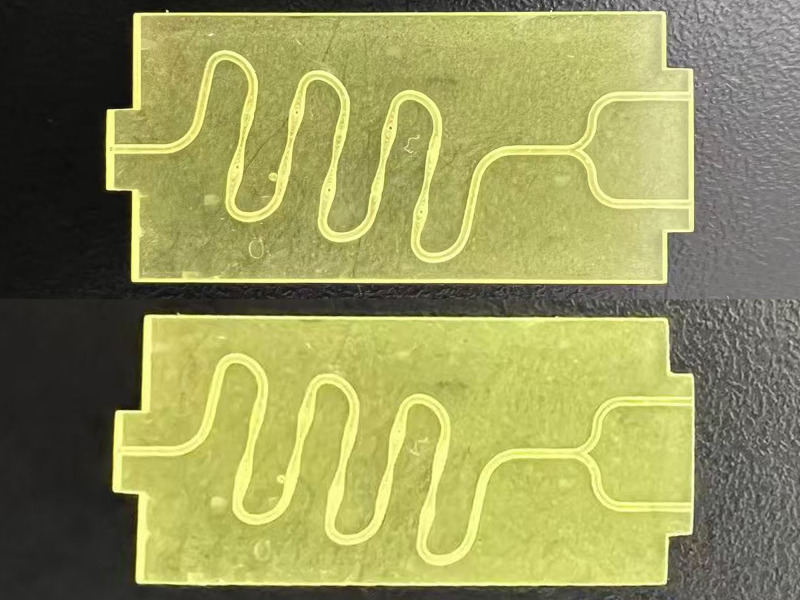
Physical image of microfluidic chip.

As shown in Fig 8, the continuous “S” type microfluidic chip flow channel structure is clearly visible, and no bubbles or chip bending occurred. However, the chip at the bottom was affected by the unpolished backside, which would have certain impacts on the subsequent microscopic observation. Therefore, the chip at the top was selected for the subsequent experiments.

### Microfluidic chip sectional characterization observation

#### Microfluidic chip section observation equipment.

**Fig 9:**
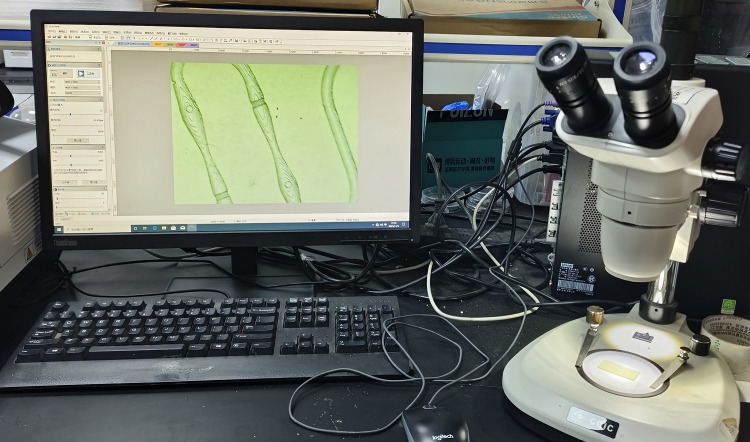
Chip Section Observation System.

As shown in Fig 9, the observation equipment selected is the COIC 1200CJS computer-type upright optical microscope produced by Chongqing Aotu Optical Instrument Co., Ltd. The microscope is connected to the computer screen and observations are conducted using the Uop View software.

#### Steps for observing the cross-section of microfluidic chips.

To visually observe the printing quality of the flow channel structure of the microfluidic chip, the three-dimensional model of the microfluidic chip is cut along the center line, and then 3D printing is carried out for preparation and sectional observation. Before the experiment began, the actual printed cross-section, surface roughness, and dimensional accuracy of the microfluidic chips were evaluated using the microscope equipment. Place the chip flat on the workbench. First, observe it at low magnification (5X) to determine the area to be examined. Then, switch to 50X for observation. After confirming that the chip printing quality basically meets the requirements, proceed with the chip section characterization observation experiment.

#### Characterization observation of the cross-section of microfluidic chips.

**Fig 10:**
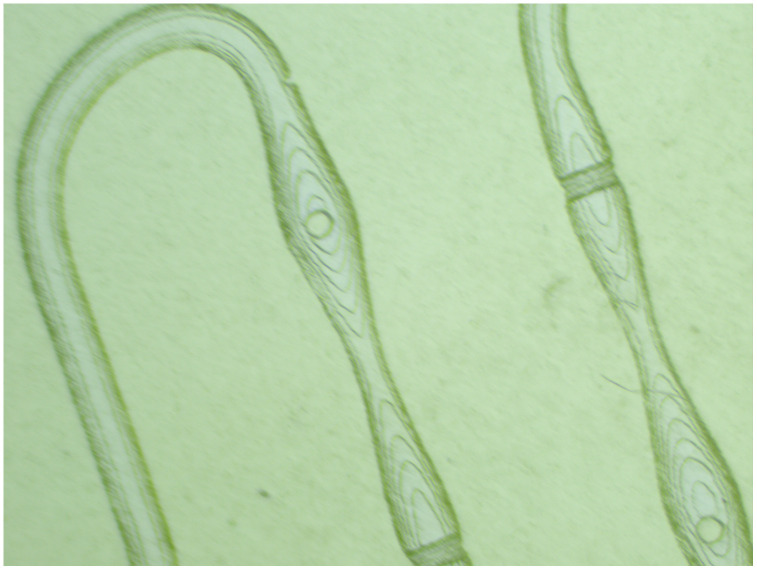
Continuous “S” shape microfluidic chip flow channel cross-section.

The six groups of bias centrifugal bends and five periodically varying linear flow channels of the continuous “S” type microfluidic chip all have a cyclic structure, with strictly consistent parameters. Therefore, the local cross-sectional diagram of the feature structure was selected for analysis. The printing situation of the chip flow channel structure is shown in Fig 10, with a printing layer thickness of 40 µm. During the bias centrifugal bend stage, the cross-sectional size of the chip flow channel remains consistent, and the printing layer transition follows the uniform characteristics of the circular cross-sectional flow channel printing. At the end of the stage, the outer baffle set can be clearly observed. In the linear flow channel stage, as the cross-sectional shape of the flow channel changes periodically, the printing layer expands radially and tangentially from the circular guide columns in the horizontal and vertical directions in a “ripple” pattern, and the printing effect of the horizontal guide column cross-section is good. The chip surface is smooth without defects, and as a whole, it meets the requirements and standards for subsequent colorimetric mixing experiments.

## Color mixing experiment

The color mixing experiment visualizes the microscopic concentration field through the mixing of different colored fluids, and is the most intuitive and reliable verification method for the mixing performance of microfluidic chips. This method not only enables qualitative observation of flow patterns and mixing interfaces, but also can achieve the evaluation of mixing efficiency under different structures and operating parameters through image analysis. The obtained analytical results also provide a visual basis for verifying the accuracy of computational fluid dynamics models, effectively supporting the research on the mixing mechanism of microfluidic chips and the optimization of design. Based on this, this study uses the existing experimental equipment to conduct color mixing experiments on microfluidic chips, combines simulation simulation cloud diagrams for comparative analysis of experimental results, and explores the actual mixing performance of microfluidic chips.

### Color mixing experiment equipment

#### Mixing equipment and reagents.

**Fig 11:**
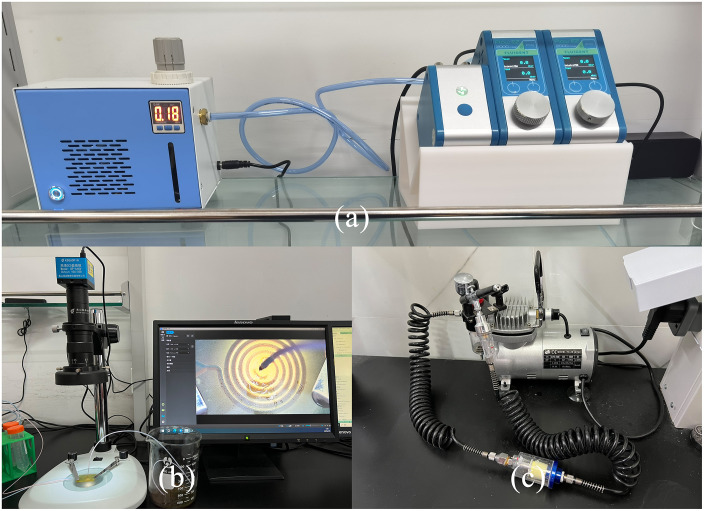
Colorimetric mixing experiment equipment. **(a)** OxyGEN system; **(b)** Electron microscope; **(c)** Air compressor.

The fluid mixing equipment is the OxyGEN system purchased from Fluigent Company, as shown in Fig 11(a). This system’s dedicated dashboard can be used as a tool for real-time control of pressure, flow rate, and valve drive in microfluidic experiments. Its modular interface is designed to be independent and simultaneously monitor all instruments and channels, specifically for the development and operation of microfluidic mixing experiments.

#### Observation equipment.

As shown in Fig 11(b), the microscope equipment used for the colorimetric mixing experiment is an electronic microscope GP-660V purchased from Kunshan Gaopin Precision Instrument Co., Ltd. The GP-660V electronic microscope is a single-tube microscope with continuous zooming capabilities. This microscope is used in conjunction with high-definition CCD and display screens; a specially designed LED lighting device is also provided, which is suitable for the observation of microfluidic chip experiments.

### Steps of the colorimetric mixing experiment

**Fig 12:**
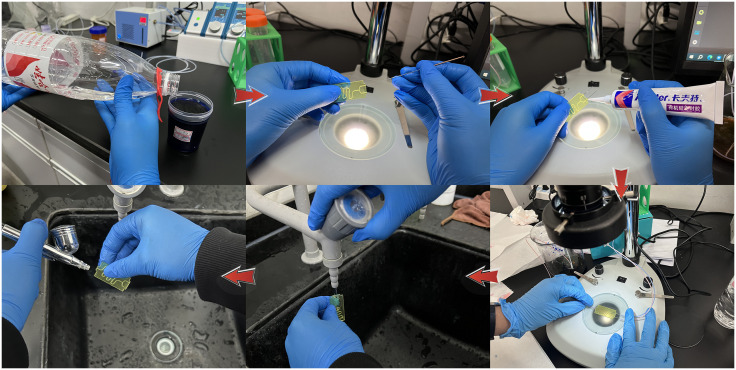
Pre- and post-processing operations for the color mixing experiment.

Before and after the actual colorimetric mixing experiment, corresponding operations need to be performed on the chips. The specific steps are shown in Fig 12.

#### Solution configuration.

Select the Diff rapid staining solution for the color comparison mixing experiment. Dilute the Diff rapid staining solution A and B with pure water at ratios of 1:40 and 1:10 respectively. After the configuration is completed, connect the two solutions to the OxyGEN system. Before the formal experiment, check the sealing of the equipment to ensure that the pressure of the injected fluid is consistent with the preset data.

#### Pre-inserting the flow channels.

Use fine probes for pre-inserting the microfluidic chip’s inlet and outlet. Slightly squeeze the inner walls of the flow channels at the inlet and outlet to avoid blockage of the channels when the probe is inserted forcefully into the chip, to prevent experimental errors caused by uneven fluid injection pressure.

#### Channel sealing.

After connecting the “experimental hose - probe - chip flow channel”, use Kafert silicone sealant to seal the connection points to prevent liquid leakage, evaporation or air entering and interfering with the flow field during the experiment. Wait for fixation before proceeding with the subsequent operations. The reasons for liquid overflow mainly include: inevitable slight angle errors when the probe is inserted forcefully into the microfluidic chip, resulting in an imperfect connection of “experimental hose - probe - chip flow channel”; as Re increases continuously, the fluid pressure values at the chip inlet and outlet gradually increase, especially at the inlet.

#### Fixing the chip.

Place the microfluidic chip connected to the OxyGEN system on the observation platform, adjust and fix the height and position of the microfluidic chip under the GP-660V electron microscope to obtain the best observation field and experimental imaging in the display. After determining the chip position, fix the microfluidic chip with the fixing clips on both sides of the observation platform. After completing the above operations, the color comparison mixing experiment can be carried out.

#### Discharging residual liquid.

After the experiment, rinse the chip with running water for 3 minutes immediately. When there is no obvious colored residue in the chip, use alcohol for re-rinsing to ensure that the experimental reagents in the chip are washed clean without residue.

#### Blowing dry the chip.

After cleaning the chip, use an air compressor to blow air into the inlet and outlet for 2 minutes (each inlet needs to be blown). To remove the alcohol in the chip, this is done to ensure that the experimental reagents in the chip are washed clean without residue. Fig 11(c) shows the 750-30L model silent air compressor used in the experiment.

### Colorimetric mixing experiment analysis

#### Continuous “S” shape microfluidic chip colorimetric mixing experiment analysis.

**Fig 13:**
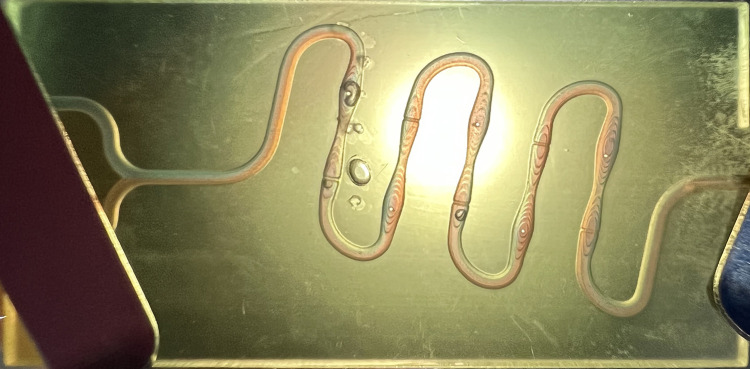
Continuous “S” type microfluidic chip mixing experiment diagram, Re = 10.

To verify the actual mixing effect of the continuous “S” shape microfluidic chip, colorimetric mixing experiments were conducted on chips under different initial conditions. Here, the mixing situation of the continuous “S” shape microfluidic chip under Re = 10 condition is analyzed, as shown in Fig 13.

When Re = 10, the two fluids show an overall trend of “unmixed - micro-mixed - basic mixed - fully mixed” within the continuous “S”-shaped microfluidic chip. At the inlet stage, the fluids present a clearly distinguishable state under the influence of laminar flow effect, and at this time, the fluids have not undergone effective mixing. When the first bias centrifugal bend is reached in the flow path, the proportion of the blue and red distinguishable areas begins to decrease, and the color of the fluid in the middle of the channel begins to show transitional areas, indicating that the two fluids start to mix from the contact surface after entering the “S”-shaped flow channel structure. At the same time, the proportion of the blue area at the end of the centrifugal bend significantly increases. The reason for this phenomenon is that the fluid generates Dean flow in the bend. Under the influence of centrifugal force, the high-speed fluid is ejected from the center of the flow path to the outside, due to the incompressibility of the fluid and the limitation of the flow path, the fluid on the outside of the bend can only loop inward along the top and bottom of the flow path, generating a secondary Dean flow, resulting in an increase in the blue area [36,37]. The phenomenon of the proportion of the single fluid color increasing at the next 5 bias centrifugal bends is similar. Above and near the chip surface directly above the first linear flow channel with periodic diameter variation, there are two groups of bubble defects, but they do not affect the mixing process of the fluid in the flow path, so the chip mixing situation can be further analyzed. When the second linear flow channel with periodic diameter variation is reached in the flow path, the proportion of the blue and red distinguishable areas further decreases, and the proportion of the transitional area significantly increases, indicating that the mixing degree of the fluid has been further improved. According to the conservation of mass equation, the fluid increases its velocity as the flow path contracts and expands, and is affected by the pressure gradient and the transverse longitudinal guide columns, the stable flow field morphology of the fluid is disrupted, inducing chaotic convection to improve the mixing efficiency. When the fourth linear flow channel with periodic diameter variation is reached in the flow path, the main part of the flow path shows a uniform light red color, and the light blue area runs in a strip along the linear flow path, indicating that the fluid has achieved basic mixing in this stage. At the 6th bias centrifugal bend to the outlet stage, the flow path shows a uniform color, the light blue area gradually disappears after the centrifugal bend, indicating that the fluid has achieved sufficient mixing when it reaches the outlet.

#### Comparative analysis of mixing performance of continuous “S”-shaped microfluidic chips.

**Fig 14:**
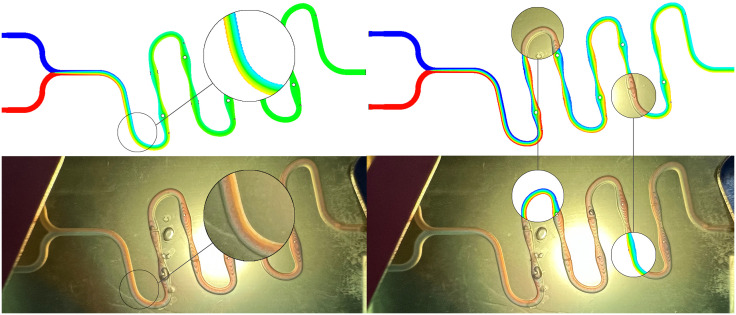
Shows the comparison between the continuous “S” type microfluidic chip simulation simulation and the hybrid experiment. The upper part is the simulation cloud diagram. The two leftmost figures have Re = 1, while the two rightmost figures have Re = 100.

As shown in Fig 14, the simulation and colorimetric mixing experiments of the continuous “S”-shaped microfluidic chips under the conditions of Re = 1 and Re = 100 are analyzed. Under the condition of Re = 1, the fluid is analyzed in detail at the position of the first bias centrifugal bend in the bend. In the local simulation diagram, the main part of the flow path shows a gradually expanding transitional area, with a uniform green center and only a small amount of red and blue areas on the left and right sides, which gradually decrease in proportion as the fluid moves. In the local colorimetric mixing experiment diagram, the color of the flow path in the middle remains uniformly stable, and the red and blue areas on the left and right sides gradually decrease in proportion as the fluid moves. The mixing degree of both is highly consistent, and the matching degree of the mixing situation is also high. Selecting the 2nd bias centrifugal bend under the condition of Re = 100 and the second half of the 5th linear flow channel with periodic diameter variation in the continuous “S”-shaped microfluidic chip for alternate analysis. At the 2nd bias centrifugal bend, in the local simulation diagram, the fluid shows a clearly distinguishable state, with a small proportion of deep red and deep blue on the inner and outer sides of the centrifugal bend, and a “yellow-green-blue” transitional area from the inside to the outside in the middle of the flow path. In the local colorimetric mixing experiment diagram, the red and blue areas occupy the main positions on the inner and outer sides of the centrifugal bend, and the middle of the flow path shows a thinner transitional strip area. At this time, both have undergone preliminary mixing, and the mixing situation is relatively consistent. In the second half of the fifth linear flow channel with periodic diameter variation, in the simulation model of the local diagram, the main part in the middle of the flow channel presents a relatively uniform green transitional area. The two sides of the flow channel are respectively thinner red and blue strip-like areas, and the degree is significantly weaker than that at the second offset centrifugal bend. In the local diagram of the colorimetric mixture experiment, the left side of the flow channel presents a thinner blue strip-like area, and red occupies the main area of the flow channel, and the degree gradually deepens from left to right until it connects with the blue strip-like area. At this time, both have achieved a basic mixture, and the mixing state is relatively consistent. In conclusion, when Re = 100 and Re = 1, the continuous “S” type microfluidic chip has a high consistency in the simulation model and colorimetric mixture experiment, and when Re = 1, it has excellent mixing performance.

## Copper ion detection experiment

### Introduction to ICP-MS technology

The ICP-MS technology has unique advantages such as extremely low detection limit, wide linear dynamic range, the ability to conduct rapid synchronous analysis of multiple elements, and the provision of isotope information. It is the key technical platform used in this study for copper ion detection experiments, for data verification of the microfluidic chip’s mixing performance [38]. Compared to the visual mixing effect provided by colorimetric mixing experiments, the quantitative data obtained from this technology also provides a crucial experimental basis for verifying the actual mixing performance of the microfluidic chip, fundamentally supporting the research on the chip’s mixing mechanism and optimization design. Based on this, this chapter conducts copper ion detection experiments on continuous “S” type microfluidic chips using inductively coupled plasma mass spectrometry technology, setting a control group to further verify the structural rationality and micro-mixing performance of the microfluidic chip.

### Equipment for copper ion detection experiment

**Fig 15:**
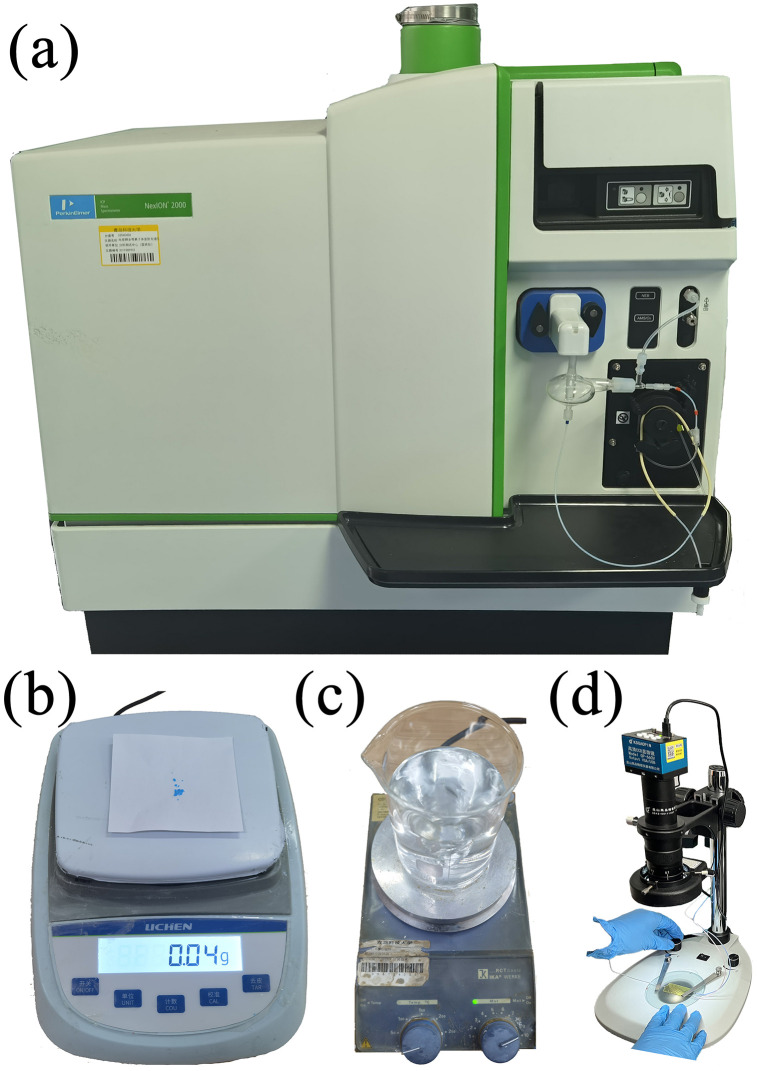
Experimental equipment for copper ion detection. **(a)** Inductively coupled plasma mass spectrometer; **(b)** Electronic balance; **(c)** Magnetic stirrer; **(d)** Mixing and observation system.

As shown in Fig 15(a), the copper ion detection equipment is an inductively coupled plasma mass spectrometer purchased from the United States PerkinElmer company, model: Nexion 2000. The inductively coupled plasma mass spectrometer is a high-sensitivity and high-precision element analysis technology that combines the high-temperature ionization characteristics of inductively coupled plasma (ICP) with the rapid multi-element detection capability of mass spectrometry (MS), suitable for detecting trace-level ions such as Cu, Hg, and As in liquids.

### Verification principles

To verify the rationality of the microfluidic chip structure and its micro-mixing performance, and to address the issue of insufficient data from the mixing experiments, we conducted two sets of control experiments to measure the concentration of copper ions in the mixed solution of the microfluidic chip. In the first set of control experiments, when magnetic stirring was applied, the solution was in a rotating state and the temperature was relatively stable. Based on the assumption that the distribution of copper ions was uniform after sufficient magnetic stirring, the average concentration data of this group was selected as the reference value. The second set of control experiments subjected the solution to static treatment to verify the influence of factors such as sedimentation stratification and natural cooling on the detection of copper ion concentration.

According to the prediction, the solution with the microfluidic chip introduced can achieve good mixing effects, and the concentration of copper ions should be uniformly distributed in the solution. The detection results should be basically consistent with the copper ion concentration in the first set of control experiments. That is to say, the experimental data of the target sample is closer to the first set of control experiments, and there is a significant difference from the second set of control experiments, which indicates that the mixing effect of the microfluidic chip is more excellent.

### Copper ion detection experiment

#### Pre-treatment.

**Fig 16:**
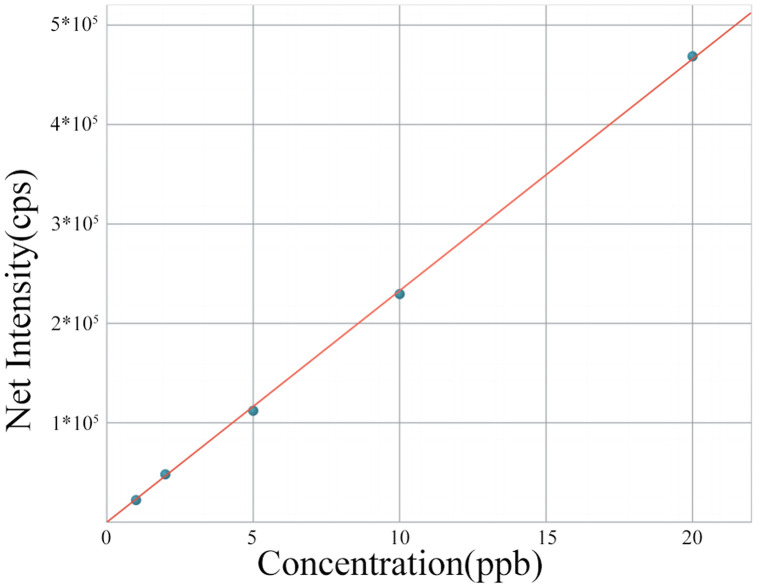
Standard Curve of Copper Ion Concentration.

Before the formal test, prepare the standard solution and draw the detection standard curve for copper ion. Take 1, 2, 5, 10, and 15 ppb as the test sample points for the curve concentration, as shown in Fig 16. After systematic analysis, the correlation coefficient is 0.999928, indicating that the linear correlation degree between the detection solution concentration and the standard curve is highly consistent, meeting the detection requirements for the subsequent experiments.

Given the high purity detection requirements of ICP-MS for the analytical solution, in this experiment, pure water was selected as the matrix, and a highly pure (over 97%) pentahydrate copper sulfate was added to prepare the sample solution to be tested. As shown in Fig 15(b), 0.04 grams of pentahydrate copper sulfate solid sample was weighed using an electronic balance and placed in a beaker, and 500 milliliters of pure water was added. As shown in Fig 15(c), the sample was continuously stirred with a magnetic stirrer for 30 minutes until no solid residue remained, and then the stirring was continued for another 30 minutes. A certain amount of the solution was taken and diluted with an equal amount of pure water multiple times to meet the detection concentration standard (1–10 μg/L). 15 milliliters of the solution was taken for static treatment to obtain the first sample solution (control sample); 30 milliliters of the solution was divided and filled into two storage bottles as the mixture solution, and the mixture experiment was conducted through the OxyGEN system, and then 15 milliliters was taken to obtain the second sample solution (target sample); 15 milliliters of the continuously stirred diluted solution was taken to obtain the third sample solution (reference sample).

#### Experimental testing and data analysis.

Using the inductively coupled plasma mass spectrometer, the three samples obtained were immediately tested. To ensure the accuracy and repeatability of the experimental data, three sets of solutions were taken from each of the three samples for testing, and the obtained experimental data were averaged. According to the verification principle in this article, it is known that the copper ion distribution in the third sample solution is uniform by default. Therefore, the average concentration data of this group was selected as the benchmark.

As shown in Table 4, the difference of the second group of solutions (the target sample) is 0.029 ppb, and the ratio reaches 101.99%. The difference between this solution and the average concentration of the third group is controlled within 2%. Although the experimental steps have controlled the variables, considering that there may be experimental conditions or other hidden factors affecting the results during the experiment, these data can still prove that the microfluidic chip has achieved the effect of fully mixing the two liquids, thereby verifying the rationality of the chip structure design. The difference of the first group of solutions (the control sample) is 0.224 ppb, which is 7.72 times that of the second group, and the ratio is 13.36 percentage points higher. The possible reason for this might be that this group remained stationary for a long time, causing copper ions to slowly settle down, or the solution temperature was slightly lower than that of the other two groups, resulting in uneven distribution of metal ions and thereby affecting the sampling concentration.

**Table 4:** Three sample solutions used for comparing copper ion concentrations.

Group	Single concentration (ppb)	Average concentration (ppb)	Difference Value (ppb)	Ratio (based on magnetic stirring, %)
Chip Integration	1.486	1.488	0.029	101.99
	1.485			
	1.493			
Magnetic Stirring	1.459	1.459	0	100
	1.454			
	1.463			
Static Treatment	1.695	1.683	0.224	115.35
	1.681			
	1.673			

## Conclusion

This study successfully designed and verified a new type of passive micro-mixing chip based on an asymmetric variable cross-section structure. The research content included chip structure design, simulation modeling, chip fabrication, mixing experiments, and copper ion experiments, totaling 5 steps. By setting up 8 groups of different Reynolds numbers for simulation, the microfluidic chip achieved the optimal quality fraction mixing index under the Re = 0.5 condition, with a numerical value of 0.9998 and a corresponding pressure drop value of 0.1502 Pa. In the colorimetric mixing experiment, the mixing performance of the microfluidic chip was stable and highly consistent with the simulation results, especially under low Reynolds numbers, achieving efficient mixing beyond conventional mixers. The chemical detection experiment indicated that under sufficient magnetic stirring conditions, the copper ion concentration in the mixed solution of the microfluidic chip approached the original concentration of the solution, fully verifying its excellent mixing performance. Through visual colorimetric mixing experiments and data-based copper ion detection experiments, the comprehensive mixing effect and fluid flow characteristics of the continuous “S” type microfluidic chip were deeply discussed and analyzed, verifying the actual mixing performance of the chip. Therefore, this microfluidic chip demonstrated excellent mixing effects both in theory and practice, and the two mixing effects had a high degree of consistency, providing a reference for promoting the practical application of passive microfluidic chips in the field of marine heavy metal detection.
